# Phytochemical Profiling and Antibacterial Activity of Methanol Leaf Extract of *Skimmia anquetilia*

**DOI:** 10.3390/plants11131667

**Published:** 2022-06-23

**Authors:** Masarat Nabi, Mohammed Iqbal Zargar, Nahida Tabassum, Bashir Ahmad Ganai, Shahid Ud Din Wani, Sultan Alshehri, Prawez Alam, Faiyaz Shakeel

**Affiliations:** 1 Department of Environmental Science, University of Kashmir, Srinagar 190006, Jammu & Kashmir, India; masaratnabi@gmail.com; 2 Department of Pharmaceutical Sciences, University of Kashmir, Srinagar 190006, Jammu & Kashmir, India; iqbalzargar@gmail.com (M.I.Z.); nahida@uok.edu.in (N.T.); shahidpharma2013@gmail.com (S.U.D.W.); 3 Center of Research for Development, University of Kashmir, Srinagar 190006, Jammu & Kashmir, India; 4 Department of Pharmaceutics, College of Pharmacy, King Saud University, Riyadh 11451, Saudi Arabia; salshehri1@ksu.edu.sa (S.A.); fsahmad@ksu.edu.sa (F.S.); 5 Department of Pharmacognosy, College of Pharmacy, Prince Sattam Bin Abdulaziz University, Al-Kharj 11942, Saudi Arabia; prawez_pharma@yahoo.com

**Keywords:** antibacterial activity, bioactive compounds, GC-MS, *Skimmia anquetilia*, Western Himalaya

## Abstract

*Skimmia anquetilia* is a plant species native to the Western Himalaya region that has tremendous potential for phytochemical activities. This study aimed to identify bioactive compounds and assess the antibacterial activity of *S. anquetilia*. To determine the major bioactive chemicals in the methanol leaf extract of *S. anquetilia*, we used a gas chromatography–mass spectrometer (GC-MS). The presence of 35 distinct phytoconstituents was discovered using GC-MS, which could contribute to the therapeutic capabilities of this plant species. The most predominant compound was 2R-acetoxymethyl-1,3,3-trimethyl-4t-(3-methyl-2-buten-1-yl)-1t-cyclohexanol (23.9%). Further, the leaf extract was evaluated for antibacterial activity against *Pseudomonas aeruginosa*, *Escherichia coli*, *Klebsiella pneumoniae*, *Salmonella typhi*, and *Staphylococcus aureus*. The extract showed the highest zone of inhibition against *E. coli* (19 mm) followed by *P. aeruginosa* (18 mm) and *K. Pneumoniae* (17 mm) at 160 mg mL^−1^. The minimum inhibitory concentration (MIC) of methanol extract against the strain of *P. aeruginosa* (2 mg mL^−1^) demonstrated significant antibacterial activity. The findings of the present study highlight the potential of *S. anquetilia* for the development of herbal medicines for the treatment of various pathogenic infections.

## 1. Introduction

The increasing interest in medicinal plants instigates from their widespread use in conventional medicines, particularly in developing nations [1]. Contemporary synthetic drugs are often investigated indecisively because they have adverse effects, whereas herbal medicines attract tremendous attention as they are more sustainable, safe, environmentally sound, and free of side effects [2]. Most medicinal plants are unique in their ability to treat and cure various human ailments due to the involvement of numerous useful phytocompounds present in diverse plant components [3]. Various medicinal plants have been employed as traditional remedies in India’s indigenous medicine systems for the treatment of various ailments since ancient times [4]. Currently, about 25% of active principles have been found in medicinal plants that are used as prescription medication items [5]. According to certain studies, indigenous folk and traditional medicine systems have roughly 25,000 plant-specific formulations that are suggested by approximately 1.5 million practitioners for preventative, persuasive, and curative purposes [6].

Important pharmaceutical functions such as anticancerous, antianalgesic, antimicrobial, antiviral, and antioxidant activities are due to the presence of numerous bioactive constituents in medicinal plants [6,7]. Raw plant extracts containing a composite blend of several phytoconstituents are widely used to make plant-based medicines. Such phytoconstituents possess distinctive and composite mechanisms and are intended to treat both long-lasting and infectious diseases [8]. In different plant species, there is a vast reservoir of bioactive compounds, however, only a small section of them have been assessed and continued to be a vital supply of bioactive entities [9]. The implementation of appropriate screening techniques is extremely significant in the quest for novel constituents as well as for quality management [10]. The extraction and structural elucidation of various such bioactive constituents from a variety of medicinal plants has contributed to the supply of some medicinal products with higher potential and quality [11]. Key information on chemical and biological activities is obtained by preliminary analysis of medicinal plants using methods such as spectrometry and chromatography, which enables the identification of bioactive plants [12]. The technique, gas chromatography–mass spectrometry (GC-MS), has been widely used in recent years to identify numerous biologically active compounds found in medicinal plants [9]. Alcohols, alkaloids, nitro-compounds, long-chain hydrocarbons, organic acids, steroids, esters, amino acids, and other bioactive phytoconstituents can all be identified using GC-MS, which requires only a small amount of plant extract [13].

*Skimmia anquetilia* is a perennial, aromatic, gregarious wild ornamental shrub native to the Western Himalaya [14]. It belongs to the family Rutaceae [15]. The essential oil of the leaves of *S. anquetilia* possesses linalool, geraniol, β-myrcene, linalyl acetate, umbelliferone, etc., and the plant has been traditionally used to treat headache, smallpox, fever, cold, rheumatism, swelling, and so on [16]. The potentialities of bioactive constituents for their use to treat various diseases must be evaluated. Therefore, the present study is focused on identifying significant functional groups and bioactive constituents from methanolic leaf extract from *S. anquetilia via* the GC-MS technique and to assess its antibacterial potential.

## 2. Results

### 2.1. Phytochemical Screening

Phytochemical screening of *S. anquetilia* leaf extract revealed the presence of various secondary metabolites, which are listed in Table 1. The phytochemical screening revealed the presence of nearly all of the phytoconstituents studied here, such as alkaloids, cardiac steroidal glycosides, flavonoids, proteins, and amino acids. However, the findings of certain tests were inconsistent. The ninhydrin test showed no proteins and amino acids in the extract, but the xanthoproteic test did. Similarly, the alkaline reagent test and lead acetate tests revealed the existence of flavonoid content in the extract, but the Shinoda test revealed the absence of flavonoid content. Tannins and carbohydrates were not detected in the extract.

### 2.2. GC-MS Analysis

**Figure 1:**
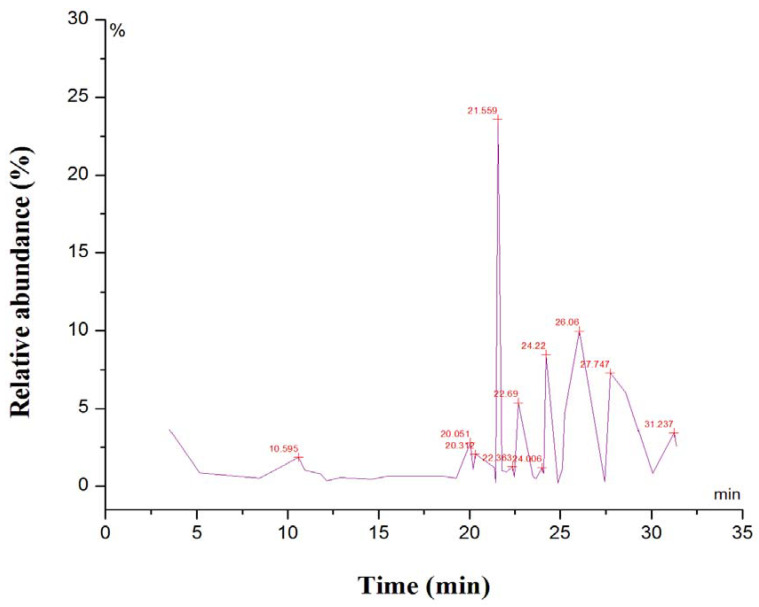
Representative GC-MS chromatogram for major compounds of methanolic leaf extract of *Skimmia anquetilia*.

The representative GC-MS spectra of the methanolic leaf extract of *S. anquetilia* is presented in Figure 1. The details about different phytochemicals identified by GC-MS with appropriate reverse search matching (RSI) are presented in Table 2. As per National Institute of Standards and Technology (NIST) library guidelines, values between 800–900 are considered a good match, and 900 or above are considered an excellent match. In the present study, the RSI values ranged from more than 800 and 900. The chromatogram of methanol leaf extract of *S. anquetilia* recorded a total of 35 peaks corresponding to the bioactive compounds that were recognized by relating mass spectral fragmentation patterns to that of the known compounds illustrated by the NIST library. The bioactive compounds in the methanolic leaf extract of *S. anquetilia* were detected to be: 1,3,5-cycloheptatriene, 2-propenoic acid, butyl ester, geijerene, linalyl acetate, linalool, glycerol 1,2-diacetate, methyl (2E,5E)-2,5-octadecadienoate, geranyl acetate, 3,7,11-trimethyl-3-hydroxy-6,10-dodecadien-1-yl acetate, 3-hydroxypropanoic acid 1-butyl ester, hexadecanoic acid, methyl ester, 2H-1-benzopyran-2-one, 5,7-dimethoxy-, 7H-furo [3,2-g][1]benzopyran-7-one, 4-methoxy-, 5,10-pentadecadienal, (Z,Z)-, photocitral A, 2H-1-benzopyran-2-one, tetradecanoic acid, 7-methoxy-6-(3-methyl-2-butenyl)-, 2R-acetoxymethyl-1,3,3-trimethyl-4t-(3-methyl-2-buten-1-yl)-1t-cyclohexanol, 1,3,3-trimethyl-2-hydroxymethyl-3,3-dimethyl-4-(3-methylbut-2-enyl)-cyclohexene, pentanedioic acid, 2,2-dimethyl-, dimethyl ester, isoauraptene, 10-pentadecen-5-yn-1-ol, (E)-, 2-phenyl-4-ethyl-oxadiazol-1,3,4-one-5, nonacos-1-ene, 2,6,10,14-tetramethylpentadecan-6-ol, 8-(2,3-dihydroxy-3-methylbutyl)-7-methoxy-2H-chromen-2-one, clionasterol acetate, wampetin, (E)-, squalene, (3E,5E,7E)-6-methyl-8-(2,6,6-trimethyl-1-cyclohexenyl)-3,5,7-octatrien-2-one, cyclohexene, 1,5,5-trimethyl-6-(2-propenylidene)-, ergost-5-en-3-ol, (3ß)-, 2-isopropyl-5-methylcyclohexyl 3-(1-(4-chlorophenyl)-3-oxobutyl)-coumarin-4-yl carbonate, and 6ß-hydroxy-17-oxo-4,5-secoandrostan-4-oic acid.

### 2.3. Antibacterial Activity

**Figure 2:**
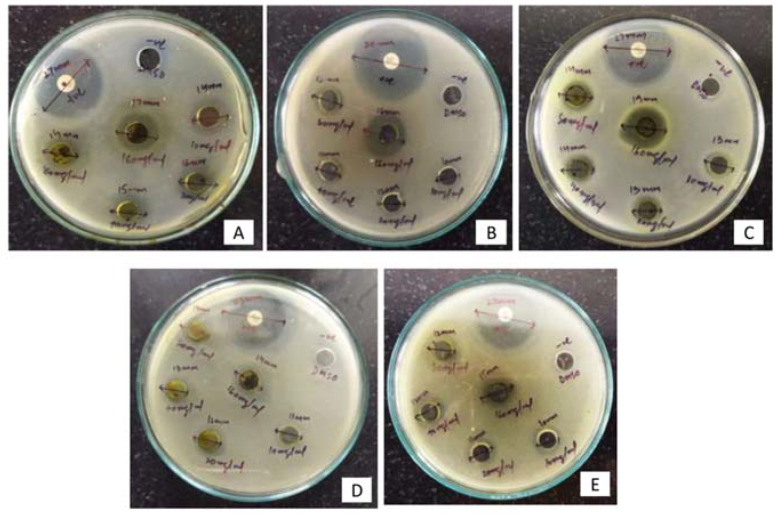
The inhibition zones (mm) of methanol leaf extract of *Skimmia anquetilia* against (**A**) *Escherichia coli* (**B**) *Pseudomonas aeruginosa* (**C**) *Klebsiella pneumoniae* (**D**) *Salmonella typhi*, and (**E**) *Staphylococcus aureus*.

The results of antibacterial activity of methanolic leaf extract are presented in Table 3 and Figure 2. The highest zone of inhibition of 19 mm was recorded against *E. coli* followed by 18 mm and 17 mm against *P. aeruginosa* and *K. pneumoniae*, respectively, at 160 mg mL^−1^ compared with other tested concentrations. The methanol extract showed a minimum inhibitory concentration against *P. aeruginosa* with a MIC of 2 mg mL^−1^.

## 3. Discussion

In the present investigation, the methanolic leaf extract of *S. anquetilia* revealed the presence of a wide variety of bioactive compounds, including flavonoids, alcohols, carbohydrates, anthocyanin, cardiac glycosides, tannins, furocoumarins, fatty acids and esters, aldehydes, amino acids, alkanes, alkaloids, steroids, terpenoids, phytosterols, saponins, alkenes, ketones, monoterpenes, and diterpenes etc. These phytocompounds could be responsible for the therapeutic capability of the methanol leaf extract of *S. anquetilia*. The chemical, 2R-acetoxymethyl-1,3,3-trimethyl-4t-(3-methyl-2-buten-1-yl)-1t-cyclohexanol, isolated from marine *Streptomyces* sp. VITJS8, has been reported for its antibacterial, antioxidant, and anticancer activities [17]. 1,3,5-Cycloheptatriene has been reported to be present in the chloroform root extract of endophytic fungus *Aspergillus terreus* var. *aureus*, exhibiting antitumor activities against HepG2 and Hep-2 cell lines, antioxidant activity, and antimicrobial activity against *Escherichia coli*, *Staphylococcus aureus*, and *Kelibsiella* sp. [18]. The phytoconstituent 1,3,3-trimethyl-2-hydroxymethyl-3,3-dimethyl-4-(3-methylbut-2-enyl)-cyclohexene has been identified in ethanol/methanol wood extract of *Populus lasiocarpa* [19] and essential oils of *Cymbopogon winterianus*, *Cymbopogon martini*, and *Pogostemon cablin*, exhibiting insecticidal properties [20]. Linalyl acetate is a monoterpene, has been the main constituent of various essential oils known to possess numerous bio-efficacies, for instance anti-inflammatory activity [21], anti-psoriatic activity [22], etc. A furocoumarin compound, wampetin, known to possess anti-inflammatory activity [23] was isolated from the twigs and roots of *Clausena lansium* [24]. The (3E,5E,7E)-6-methyl-8-(2,6,6-trimethyl-1-cyclohexenyl)-3,5,7-octatrien-2-one has been identified in ethanolic leaf extract of *Passiflorain carnata* and displayed antitumor activity using an *in-silico* approach [25]. Similarly, 2-isopropyl-5-methylcyclohexyl 3-(1-(4-chlorophenyl)-3-oxobutyl)-coumarin-4-yl carbonate is a benzopyrone, and 1-hexyl-2-nitro cyclohexane is a ketonic compound and both have been identified from petroleum ether leaf extract of *Crateva adansonii* and revealed anti-inflammatory properties by using combined analysis *viz.* *in-vitro* phytochemical and *in-silico* studies [26]. Hexadecanoic acid, methyl ester is a fatty acid and has been reported to possess multiple bioactivities such as antioxidant, anticoronary, hepatoprotective, antiacne, anti-inflammatory, antiarthritic, anticancer, antihistaminic, antieczemic, α-reductase inhibitory, and antiandrogenic activities [27]. Likewise, the coumarin compound 7H-furo [3,2-g][1]benzopyran-7-one, 4-methoxy, also known as bergapten, has been isolated from the methanol root extract of *Zanthoxylum flavum* and exhibited significant antimalarial activity [28]. On the other hand, an aldehydic polar compound, photocitral A, has been extracted from methanol leaf extract of *Kalanchoe pinnata* and documented with moderate interaction affinity for ion channels, nuclear receptors, and enzyme inhibition activity [29].

From the evidence presented above, it is clear that the *S. anquetilia* plant contains a variety of pharmacological constituents with important pharmacological properties such as antimicrobial, antioxidant, antitumor, anti-inflammatory, antidiabetic, anticoronary, analgesic, antiaging, anticancer, antipsoriatic, hepatoprotective, hypercholesterolemic, antihistaminic, antiandrogenic, diuretic, and antieczemic, etc. As a result, the discovery of numerous phytochemical components from *S. anquetilia*’s methanol leaf extract demonstrates the plant’s important therapeutic qualities. Bio-prospecting and other investigations are needed to support the biological qualities and biological value of these new biomolecules.

## 4. Materials and Methods

### 4.1. Chemicals and Reagents

All chemicals and reagents used were of analytical grade. Methanol, nutrient agar, Mueller–Hinton agar (MHA) and dimethyl sulfoxide (DMSO) were purchased from E-Merck (Mumbai, India). Gentamycin was procured from Sigma Aldrich (St. Louis, MO, USA).

### 4.2. Collection and Identification of Plant Material

*Skimmia anquetilia* N.P. Taylor and Airy Shaw used for the investigation was obtained from the Gulmarg area (74°22′40.01″ N, 34°03′02.26″ E) of the Baramulla District, Kashmir, India at an altitude of 2734 m above msl. The plant specimen was authenticated by Dr. Akhter Hussain Malik, Centre for Biodiversity and Taxonomy (CBT), Kashmir University. The voucher number is 2697-(KASH).

### 4.3. Sample Extraction

The freshly collected leaf material was thoroughly washed with running tap water and rinsed in distilled water before the leaves were cut into small pieces then dried under shade at room temperature for 15 days, then powdered using a laboratory electric blender. The dried leaf powder (80 g) was extracted using methanol solvent (800 mL) in a soxhlet extractor for 8 h at a temperature below the boiling point of the solvent. Repetitive extraction of the plant material was carried out before the attainment of colourless solvent. The extract was filtered using Whatman No. 1 filter paper, and the acquired filtrate was then evaporated to dryness using a rotary evaporator and stored at 4 °C in an airtight glass container for further analysis [30]. The yield was about 22%.

### 4.4. Determination of Plant Extract Yield (%)

Yield percentage (%*w*/*w*) of the dried extracts was calculated as:

Yield (%)=W1×100/W2

where W1 is the dry weight of extract after solvent evaporation and W2 is the weight of the dried leaf powder.

### 4.5. Qualitative Phytochemical Analysis

The methanol leaf extracts of *S. anquetilia* were subjected to qualitative analysis for secondary metabolites including alkaloids, tannins, flavonoids, cardiac steroidal glycosides, proteins and amino acids, and carbohydrates according to the standard methods discussed in the literature [31].

### 4.6. GC-MS Analysis

Analysis of the leaf extract of *Skimmia anquetilia* was conducted using the Thermo scientific “Chromeleon” (c) Dionex Version: 7.2.8.10783 (Agilent technologies, Santa Clara, CA, USA) instrument. Helium gas (99.99%) was used as the carrier gas with a flow rate of 1 mL min^−1^. GC-MS investigation was performed by employing the following conditions: high electron ionization energy (70 eV) was used. Initially furnace temperature was maintained at 60 °C, then raised to 160 °C with a 4 °C min^−1^ increasing rate and a holding time of approximately 10 min. The temperature was eventually raised at 10 °C min^−1^ to 350 °C. Then, 1 mL of the sample was kept in a 2 mL screw-top vial in an autoinjector, and 1 μL of the sample was injected in split mode (1:40). The overall run time of the GC was 33 min.

#### Identification of Compounds

The compounds were identified based on mass spectral patterns with those spectral databases of compounds stored in the NIST electronic library coupled with the GC-MS system. The spectrum of the unknown components was compared with the spectrum of known components stored in the NIST library. The name, molecular weight and molecular formula of the compounds were ascertained.

### 4.7. Antibacterial Activity

Antibacterial activity of methanol extract of *S. anquetilia* was determined through the agar well diffusion technique [32]. Tubes with 20 mL of nutrient agar media were inoculated with freshly prepared bacterial inoculums using a sterile loop in a back-and-forth motion to ensure an even distribution of inoculums. Petri plates were prepared by pouring pre-inoculated media and allowing it to solidify and then 8 mm wells were made using a sterile cork borer. A total of 100 μL of different concentrations (10, 20, 40, 80, and 160 mg mL^−1^) of extract and an equal volume of negative control dimethyl sulfoxide (DMSO) were poured into the wells. The plates were allowed to stand for 30 min to allow pre-diffusion of the extract into the medium and incubated at 37 °C for 17 h. The plates were observed for zones of inhibition after 17 h and results were compared with those of the positive control containing gentamycin (10 μg mL^−1^).

### 4.8. Bacterial Strains, Media, and Controls

Five bacterial strains *viz.*, *Pseudomonas aeruginosa* (MTCC424), *Escherichia coli* (MTCC739), *Klebsiella pneumoniae* (MTCC139), *Salmonella typhi* (MTCC3224), and *Staphylococcus aureus* (MTCC96) were used in this study; these were procured from the Institute of Microbial Technology (IMTECH), Chandigarh, India. The strains included both Gram-negative as well as Gram-positive strains; for the agar well diffusion assay, all strains were first sub-cultured in nutrient agar media and incubated at 37 °C for 18 ± 2 h. For the antibacterial assay, gentamycin (10 μg mL^−1^) was used as a positive control, whereas DMSO was used as a negative control.

### 4.9. Determination of MIC

The agar dilution method [33] was used to determine the antibacterial potential of the methanol extract. A two-fold serial dilution of the extract at varying concentrations from 32 to 0.25 mg mL^−1^ was prepared in Mueller–Hinton agar (MHA) at 48 °C. Plates were dried at room temperature for 30 min prior to spot inoculation with a loopful of an optimized concentration of each bacterial strain (10^8^ CFU mL^−1^) using the 0.5 McFarland standard. The negative control included a blank plate containing only MHA. The plates were incubated at 37 °C for 18 h. Experiments were performed in triplicate, the agar plates were observed and compared with the growth in the negative control for their visible bacterial growth both pre- as well as post-incubation. The Minimum inhibitory concentrations (MICs) were determined as the least concentration of extract inhibiting any noticeable growth of each organism on the agar plates.

## 5. Conclusions

In the present study, *Skimmia anquetilia* leaves have shown to have various secondary metabolites that possess many pharmacological properties of which antibacterial activity is one. The GC-MS analysis showed the presence of 35 distinct bioactive compounds which contribute to activities such as antifungal, antibacterial, antioxidant, anticancer, antidiabetic, antipsoriatic, anti-inflammatory, and so on. Therefore, the presence of phytochemicals is responsible for the different therapeutic and pharmacological effects. For the development of novel medications based on the bioactive chemicals identified in *S. anquetilia*, more research is needed to assess its bioactivity, toxicity profile, and clinical investigations.

## Figures and Tables

**Table 1:** Phytochemical screening of methanol leaf extract of *Skimmia anquetilia*.

Phytoconstituent	Name of the Assay	Methanol Extract
Alkaloids	Mayer’s test	+
	Wagner’s test	+
	Dragendorff’s test	+
Carbohydrates	Benedict’s test	−
Cardiac steroidal glycosides	Keller–Kiliani test	+
Flavonoids	Shinoda test	−
	Alkaline reagent test	+
	Lead acetate test	+
Proteins and amino acids	Xanthoproteic test	+
	Ninhydrin test	−
Tannins	Ferric chloride test	−
	Gelatin test	−

+ Indicates the presence and − indicates the absence.

**Table 2:** Bioactive compounds of methanolic leaf extract of *Skimmia anquetlia* identified using GC-MS analysis.

S. N.	Retention Time (min)	Phytocompounds	CAS Number	Peak Area (%)	RSI	Molecular Formula	Molecular Weight (g/mol)
1.	3.490	1,3,5-Cycloheptatriene	544-25-2	3.76	914	C_7_H_8_	92.14
2.	5.150	2-Propenoic acid, butyl ester	141-32-2	0.86	890	C_7_H_12_O_2_	128.16
3.	8.453	Geijerene	6902-73-4	0.62	-	C_12_H_18_	162.2713
4.	10.595	Linalyl acetate	115-95-7	1.85	937	C_12_H_20_O_2_	196.29
5.	10.950	Linalool	78-70-6	1.65	858	C_10_H_18_O	154.25
6.	11.813	Glycerol 1,2-diacetate	102-62-5	0.79	941	C_7_H_12_O_5_	176.16
7.	12.152	Geranyl acetate	105-87-3	0.44	925	C_12_H_20_O_2_	196.29
8.	12.891	Methyl (2E,5E)-2,5-octadecadienoate	56846-97-0	0.64	-	C_19_H_30_O_2_	294.5
9.	14.636	3,7,11-Trimethyl-3-hydroxy-6,10-dodecadien-1-yl acetate	0	0.57	939	C_17_H_30_O_3_	282.41
10.	15.493	3-Hydroxypropanoic acid 1-butyl ester	0	0.76	819	C_7_H_14_O_3_	146.18
11.	18.489	Hexadecanoic acid, methyl ester	112-39-0	0.64	916	C_17_H_34_O_2_	270.45
12.	19.255	2H-1-Benzopyran-2-one, 5,7-dimethoxy-	487-06-9	0.61	984	C_11_H_10_O_4_	206.19
13.	20.051	7H-Furo [3,2-g][1]benzopyran-7-one, 4-methoxy-	484-20-8	2.79	942	C_12_H_8_O_4_	216.18
14.	20.204	5,10-Pentadecadienal, (Z,Z)-	64275-49-6	1.69	911	C_15_H_26_O	222.37
15.	20.312	Photocitral A	55253-28-6	2.55	948	C_10_H_16_O	152.23
16.	21.391	2H-1-Benzopyran-2-one, 7-methoxy-6-(3-methyl-2-butenyl)-	581-31-7	1.32	923	C_15_H_16_O_3_	244.28
17.	21.439	Tetradecanoic acid	544-63-8	0.33	-	C_14_H_28_O_2_	228
18.	21.559	2R-Acetoxymethyl-1,3,3-trimethyl-4t-(3-methyl-2-buten-1-yl)-1t-cyclohexanol	0	23.9	932	C_17_H_30_O_3_	282.4
19.	21.799	1,3,3-Trimethyl-2-hydroxymethyl-3,3-dimethyl-4-(3-methylbut-2-enyl)-cyclohexene	0	1.59	946	C_15_H_26_O	222.37
20.	22.027	Pentanedioic acid, 2,2-dimethyl-, dimethyl ester	13051-32-6	0.93	824	C_8_H_14_O_4_	174.19
21.	22.363	Isoauraptene	1088-17-1	1.77	889	C_15_H_16_O_4_	260.28
22.	22.462	10-Pentadecen-5-yn-1-ol, (E)-	64275-59-8	0.68	880	C_15_H_26_O	222.37
23.	23.493	Nonacos-1-ene	18835-35-3	0.62	935	C_29_H_52_	400.72
24.	23.663	2,6,10,14-Tetramethylpentadecan-6-ol	104000-14-8	0.68	892	C_19_H_40_O	284.5
25.	24.078	1-Dodecanol, 3,7,11-trimethyl-	6750-34-1	0.89	868	C_15_H_32_O	228.41
26.	24.220	8-(2,3-Dihydroxy-3-methylbutyl)-7-methoxy-2H-chromen-2-one	5673-37-0	8.47	813	C_15_H_18_O_5_	278.30
27.	25.122	10-Pentadecen-5-yn-1-ol, (E)-	64275-59-8	1.69	974	C_15_H_26_O	222.37
28.	25.254	Wampetin	89824-26-0	4.73	860	C_21_H_18_O_6_	366.4
29.	26.060	Squalene	111-02-4	9.98	933	C_30_H_50_	410.72
30.	27.445	(3E,5E,7E)-6-Methyl-8-(2,6,6-trimethyl-1-cyclohexenyl)-3,5,7-octatrien-2-one	17974-57-1	0.90	858	C_18_H_26_O	258.399
31.	27.747	Cyclohexene, 1,5,5-trimethyl-6-(2-propenylidene)-	56248-17-0	7.79	885	C_12_H_18_	162.27
32.	28.608	Clionasterol acetate	4651-54-1	6.59	888	C_31_H_52_O_2_	456.7
33.	30.057	Ergost-5-en-3-ol, (3ß)-	4651-51-8	0.85	890	C_28_H_48_O	400.7
34.	31.237	2-Isopropyl-5-methylcyclohexyl 3-(1-(4-chlorophenyl)-3-oxobutyl)-coumarin-4-yl carbonate	0	3.43	896	C_30_H_33_ClO_6_	525
35.	31.366	6ß-Hydroxy-17-oxo-4,5-secoandrostan-4-oic acid	59251-83-1	2.53	821	C_19_H_30_O_4_	322.4

Identification of compounds was carried out by GC-MS spectrum. In the GC-MS spectrum, the requisites should be that RSI values were more than 800 or 900 according to NIST library guidelines.

**Table 3:** *In-vitro* antibacterial activity and MIC of *S. anquetilia* methanol leaf extract against tested bacterial strains.

Concentration (mg mL^−1^)	Zone of Inhibition (mm) (Mean ± SD)				
	Gram-Negative Bacteria				Gram-Positive Bacteria
	*E. coli* *	*P. aeruginosa* *	*K. pneumoniae* *	*S. typhi* *	*S. aureus* **
10	13.0 ± 0.57	10.0 ± 5.18	14.0 ± 0.57	7.0 ± 6.08	10.0 ± 0.57
20	14.0 ± 0.57	12.0 ± 0.57	15.0 ± 0.57	7.0 ± 6.08	12.0 ± 1.15
40	14.0 ± 0.57	12.0 ± 0.57	15.0 ± 0.57	8.0 ± 6.65	12.0 ± 0.57
80	13.0 ± 0.57	13.0 ± 0.57	13.0 ± 0.57	9.0 ± 7.57	13.0 ± 0.57
160	19.0 ± 0.57	18.0 ± 0.57	17.0 ± 0.57	8.0 ± 6.92	16.0 ± 1.52
PC (10 µg)	26.0 ± 1.0	29.0 ± 1.0	28.0 ± 0.57	29.0 ± 1.73	28.0 ± 1.52
MIC (mg mL^−1^)	8.0 ± 0.56	2.0 ± 0.57	8.0 ± 0.55	32.0 ± 0.57	16.0 ± 0.50

Values are in triplicate and represented as mean ± SD; PC = positive control. * Gram-negative bacteria, ** Gram-positive bacteria.

## Data Availability

Not applicable.
